# Health Problems & Barriers to Healthcare Services for the Transgender Community in Lahore, Pakistan

**DOI:** 10.12669/pjms.38.1.4375

**Published:** 2022

**Authors:** Iram Manzoor, Zartasha Hanan Khan, Rafia Tariq, Rijah Shahzad

**Affiliations:** 1 Dr. Iram Manzoor, MBBS, FCPS, MSc, MCPS-HPE, PhD. Department of Community Medicine, Akhtar Saeed Medical and Dental College, Lahore, Pakistan; 2 Dr. Zartasha Khan, MBBS, MCPS Trainee. Department of Community Medicine, Akhtar Saeed Medical and Dental College, Lahore, Pakistan; 3 Dr. Rafia Tariq, 4^th^ year MBBS student. Akhtar Saeed Medical and Dental College, Lahore, Pakistan; 4 Dr. Rijah Shahzad, 4^th^ year MBBS student. Akhtar Saeed Medical and Dental College, Lahore, Pakistan

**Keywords:** Transgender, Healthcare, Barrier

## Abstract

**Objectives:**

To find out the major health problems and barriers in getting health care by transgender community in Lahore, Pakistan.

**Methods:**

An analytical cross-sectional study was conducted in transgender community of Lahore from January to October 2020. The study included 214 participants from different areas of Lahore by targeting their “gurus”. Non-probability, snow-ball sampling technique was used to collect required sample size. Data was collected by using a structured questionnaire. Data were analyzed using SPSS version 23. Results were generated in form of tables and graphs. Chi square test and Fischer’s exact test were used to find out associations between health seeking behavior with their transgender status and p value was fixed as ≤ 0.05 as significant.

**Results:**

Among total 214, 78.5% were transgender females and 21.5% were transgender male. Among the common health problems were depression (56%), anxiety (59%) and genital tract ulcers (45%). About 70% transgender seek health care from government hospitals. Among total 214 participants, 70% reported that they receive poor quality of health care. The main reasons of not getting proper care is non acceptance (20.7%), feeling ashamed (28.7%), non-availability of CNIC (44.5%) and un-affordability (6.1%). Significant association of transgender female with consultation with doctors (p=0.013), seeking care at government hospitals (p =0.038) poor experience at health care facility (0.050), neglect during medical treatment (p=0.015) and feeling of discrimination during treatment (p= 0.042).

**Conclusion:**

Transgender community face physical, mental, social and reproductive health issues. About 70% trans-genders receive poor quality of health care services. Non acceptance, feeling ashamed, non-availability of CNIC and non-affordability have been reported as major barriers in getting desired health care.

## INTRODUCTION

According to World Health Organization (WHO), “transgender people” is an umbrella term for all people whose internal sense of their gender (gender identity) and expression are different from the social expectations of their biological sex at birth.^1^ Approximately 0.3-0.5% of the global population is identified as transgender.^2^ According to 2017 Census of Pakistan, 10,418 transgender people were registered.^3^

Across the world transgender population face multiple disparities including familial rejection, high levels of stigma, discrimination, gender-based violence, marginalization and social exclusion. In a study which compared health disparities among transgender women in Los Angeles from 1998-99 to 2015-16, participants reported increased physical harassment, abuse, homelessness and lower levels of income.^4^

Transgender persons also suffer significant health disparities in multiple arenas. They are at risk of emotional and psychological abuse, physical and sexual violence, sexually transmitted infections, viral hepatitis and HIV, substance abuse, use of intravenous injections, depression, anxiety and suicidal thoughts. In a study conducted among transgender persons in Italy, prevalence of HIV, HBV and HCV infections was recorded to be 12.1%, 4.6% and 3.7% in male-to-female, and 0%, 4.0% and 8.0% in female-to-male participants.^5^ A U.S. sample of 1093 transgender persons demonstrated a high prevalence of clinical depression (44.1%), anxiety (33.2%) and stigmatization (27.5%).^6^ Transgender adolescents report higher suicide attempt rates compared with cisgender counterparts. They are internationally recognised as a population group that carries a disproportionate burden of HIV infection, with a worldwide prevalence of 20% and in Pakistan, the HIV incidence among transgender people contributes up to 17.5% of the entire HIV population.^7^

Transgender population face multiple barriers to access health care ranging from lack of provider knowledge regarding transgender issues to postponement of medical care due to discrimination from healthcare provider.^8^ In a middle-income country, transgender population is at greater risk of exclusion making them vulnerable to poor access to trans-specific care, HIV prevention and care, and mental health care. A study conducted in Malaysia showed that 54% of the transgenders adopted being a sex worker due to lack of employment opportunities and harassment at work.^9^

World Health Organization acknowledges the health needs of the transgender community and strongly advocates that all forms of stigma and discrimination, within or outside the health system, should be avoided. The judgement of the Supreme Court of Pakistan, declaring transgender people as full and equal citizens of Pakistan is a big milestone in the history of the country.^10^ At present little was known about the prevalent health issues of transgenders in Pakistan and barriers to access health care services. This study was carried out to find out the gaps in existing knowledge about disease status and barriers to access of health care in transgender community of Lahore.

## METHODS

An analytical cross-sectional study was conducted in transgender community in Lahore between January to October, 2020. Provincial representative of transgender community in Punjab assembly and an NGO working for transgender rights (Sangat) was targeted to reach the specific group. Due to the sensitivity of the issue of targeting a marginalized community, researchers followed the guidelines of Canadian Professional Association for Transgender Health (CPATH), developed in 2015 to avoid ethical issues. During the phase of developing the proposal, specific attention was given to issues of legitimacy and engagement of transgender community through their “gurus”. These gurus are leaders in specific allocated areas followed by their “chelas”. These gurus adopt chelas and are responsible for their bread and butter, allocation of funds and residential allotments. The researchers targeted gurus of different localities through Sangat (NGO) and took consent for engaging their chelas for their health assessment. Written consent was taken from every participant and confidentiality was maintained through out the process of data collection, analysis and interpretation. The team of researchers was available for providing prescription for the minor health problems and for referral where needed.

A sample size of 174 participants was generated using WHO sample size calculator of known population, keeping known population of Lahore for transgender less than 5000, 95% confidence interval and margin of error at 5% and power at 80%. To increase the power of study, sample size was increased to 214 to avoid less representation. An addition of 40 participants was done for adequate sample size generation. After getting approval from IRB (IRB no.#M-32/20/CM-22) Akhtar Saeed Medical and Dental College, data was collected from sample of 214 subjects through non-probability snow-ball sampling technique by targeting different “gurus” and their houses in different geographical areas of Lahore. Snow ball sampling technique is the most recommended sampling technique while dealing with marginalized group and sensitive topics. A written informed consent form was developed in the local language (Urdu) describing objectives of this research. Informed consent was taken before each interview by the interviewee describing every aspect of research in local language.

Data was collected irrespective of age and gender distribution of participants residing in Lahore. Those participants who were reluctant to answer and did not give consent were excluded from the study along with those who were not giving complete information. A structured questionnaire was formulated and piloted on 25 participants. After modifications, in light of pretesting, corrected questionnaire was used for data collection. Pilot testing helped us to restructure some of the questions and few questions related to sexual preferences were omitted, as researchers identified reluctance of the participants in answering those questions. Data was collected through one to one interview technique and responses were obtained by self-reporting of participants about their health issues. Data was entered and analyzed using SPSS version 23. Qualitative variables were presented in the form of frequency tables. Mean and standard deviation were calculated for quantitative variables including age. Chi square test were applied to analyze the association of gender with barriers in seeking health and Fischer’s Exact test was applied where the data was limited to less than five. P-value was fixed at less than and equal to 0.05 to make significant association between two genders.

## RESULTS

A total of 214 trans-genders participated in this study, out of which 46 (21.5%) were transgender male and 128 (78.5%) were transgender females. Mean age of the participants was 30.8±8.8 years. Only 83 (39%) were educated and 46 (21.5%) were employed. The monthly income of 91 (43%) participants was more than Pak Rs. 30,000. Cigarette Smoking was seen in 123 (57%) of the participants, 60 (28%) were IV drug abusers with 13 (6%) had needle sharing practices (Table-I).

**Table I:** Socio-demographic Profile of the participants (n= 214).

Characteristics	Frequency (n)	Percentage (%)
***Gender***		
Transgender Male	46	21.5
Transgender Female	128	78.5
***Age in years***		
15 - 24	45	21
25 - 34	118	55.1
35 - 44	34	15.8
45 - 54	13	6.1
55 - 65	4	1.9
***Educational Status***		
Educated	83	39
Un-educated	131	61
***Employment status***		
Employed	46	21.5
Unemployed	168	78.5
***Monthly income in Pakistani Rupees***		
Less than 10,000	18	8
11,000 – 20,000	18	8
21,000 – 30,000	87	41
More than 30,000	91	43
***Cigarette smoking***		
smoker	123	57
non-smoker	91	43
***IV drug abuse***		
abuser	60	28
non-abuser	154	72
***Needle sharing practices***		
yes	13	6
no	201	94

Health problems in transgender community are categorized as physical, mental, social and reproductive health problems. Physical health hazards were categorized as non communicable and communicable diseases. Results showed 21 out of 214, (9.8%) transgenders had chronic disease. Eighteen had Diabetes (8.4%) and 3 (1.4%) had hypertension. Prevalence of different communicable diseases were reported by 37 (17.3%) of the participants. Infection rate with COVID-19 was reported by 24 (11.2%). Physical disability was reported in 11(5%). A high number of transgenders 152 (71%) were screened for Hepatitis B and Hepatitis C but infection rate was relatively low. Mental health issues of this community showed that 120 (56%) had depression and 126 (59%) had anxiety. A vast majority reported verbally abused and suicidal thoughts too. Majority of the participants 191 (89%) had felt social discrimination and feeling of social isolation 73 (34%). Seventy six percent of participants reported physical abuse. Out of 214, one hundred and eighty-five (86%) were sexually active. Out of these 185, 94% were sexually active with male partners and 7 (4%) with other transgenders. Age at first sexual encounter was reported to be less than fifteen years in 120 (65%) of the participants. Twenty seven percent of the respondents reported more than 50 sexual partners. Regarding reproductive health issues, it was observed that 164 (77%) used condoms and 36 (17%) had awareness about STDs. Most common presentation was presence of genital ulcers followed by genital warts and discharge (Table-II).

**Table II:** Health problems in transgender community.

Health problems	Frequency(n)	Percentage (%)
***Physical Health***		
Non-Communicable disease	21	9.8
Diabetes	18	8.4
Hypertension	3	1.4
***Communicable disease***	37	17.3
COVID-19	24	11.2
Hepatitis B	3	1.4
Hepatitis C	4	1.9
HIV	2	0.9
Typhoid	2	0.9
TB	2	0.9
***Mental Health***		
Depression	120	56
Anxiety	126	59
Verbally abused	169	79
Suicidal thoughts	55	26
***Social Health***		
Isolated	73	34
Discriminated	191	89
Physically abused	163	76
***Reproductive health***		
Sexually active	185	86
Genital ulcers	45	21
Genital warts	6	2.8
Genital discharge	5	2.3
Use condoms	164	77
Paid for sex	158	74
Aware of STD’s	36	17
HIV screened	162	76

In transgender community, 182 (84%) got treatment from doctors. Most common health care facility reported to be utilized by the participants were government hospitals opted by 151 (70%). One hundred and seventy-five (81.8%) had faced neglected behavior while seeking medical treatment. One hundred and sixty-nine (79%) had faced discrimination by health care providers during treatment. When asked about the reasons for not opting health care services, out of total 164 transgenders, 47 (28.7%) felt ashamed of their transgender status (Table-III).

**Table III:** Assessment of Health Care access and barriers in utilization.

Variables	Frequency (n)	Percentage (%)
***Consultation for treatment***		
Doctor	182	85
Nurse	13	6.1
Hakeem	10	4.2
Homeopathic	5	2.3
Spiritual Healer	4	1.9
***Selection of medical setting***		
Government hospital	151	70
Private hospital	38	18
Self-treatment	24	11.5
Clinics	1	0.5
***Reasons for not going to health care facilities***		
Non-acceptance	34	20.7
Feeling ashamed	47	28.7
CNIC not available	73	44.5
Costly	10	6.1
***Routine health checkups in last two years***		
yes	103	48.1
no	111	51
***Neglected during medical treatment***		
yes	175	81
no	39	19
***Discrimination by healthcare provider***		
yes	169	79
no	45	21
***Lack of training of doctors for transgender treatment***		
yes	179	84
no	35	16

Bivariate analysis of health seeking behaviors and gender roles of transgender community showed significant association of transgender female with consultation with doctors (p=0.013), seeking care at government hospitals (p=0.038) poor experience at health care facility (0.050), neglect during medical treatment (p=0.015) and feeling of discrimination during treatment (p=0.042) (Table-IV).

**Table IV:** Bivariate analysis of Trans-male and Trans-females of Assessment of Health Care access and barriers.

Variables	Trans male	Trans female	p-value
***Consultation when sick***			
Doctor	36	142	0.013**
Nurse	1	10	
Hakeem	3	5	
Homeopathic	0	3	
Spiritual healer	1	1	
***Selection of medical setting***			
Government hospital	30	121	0.038**
Private hospital	8	30	
Self-treatment	2	10	
Clinics	1	0	
***Reasons for not going to healthcare facilities***			
Feeling of Non acceptance	2	10	0.378
Feeling ashamed	2	5	
CNIC not available	0	1	
Costly	0	4	
***Experience of availability to healthcare***			
Excellent	0	2	0.050**
Good	5	18	
Fair	12	29	
Poor	25	116	
***Routine health checkups in last two years***			
Yes	29	74	0.006**
No	13	89	
***Neglected during medical treatment***			
Yes	34	141	0.015**
No	8	22	
***Discrimination by healthcare provider***			
Yes	31	138	0.042**
No	11	25	
***Lack of trained doctors for transgender treatment***			
Yes	33	146	0.029**
No	9	18	

## DISCUSSION

Transgender community is a marginalized community and is at high risk of physical, social and mental health issues based on their gender identity. This particular study aimed to find out different health issues of this group along with the barriers they face during seeking help from health care providers. In this study, out of 214 transgenders, 128 (78.5%) were trans-females and 46 (21.5%) were trans-males. In another study based upon the health requirements of transgenders in Mississippi, 43% identified themselves as males while 47% reported as trans-females.^11^ The mean age of our respondents is 28 years that falls within the international bracket of 23±4.98 years, of participants of Jackson, Mississippi too. In this study, majority of the respondents (61%) were denied access to education. Formulated on the findings of a 2008 meta-analysis, 27.7% of the transgenders in the world had not completed high school which is relatively similar to our deductions.^12^ Data showed that 43% of the respondents have a monthly income of more than Rs 30000, 41% earn about Rs 20,000-30,000 while others fail to make enough money for their survival. Findings from a survey report a median average monthly income of US$150 which is equal to Rs 24414.18 in the past six months.^11^ As per this study, 57% of the respondents confessed that they smoked approximately 10-20 cigarettes per day while 28% reported to be involved in IV drug abuse. Majority of IV abusers consisting of 94% denied sharing needles as they were aware of the negative consequences associated with it. In a transgender survey conducted in Pakistan, 66.7% of the transgenders reported being smokers and a high rate of drug abuse was also reported.^13^

Physical health profiling of the participants showed that 8.4% were found diabetic and 1.4% were known cases of hypertension. In a study conducted in United States from 2014 to 2017, multivariable analysis revealed that transgender men had a >2-fold and 4-fold increase in the rate of myocardial infarction compared with cisgender men because of the presence of hypertension and diabetes as risk factors.^14^ Prevalence of infectious diseases amongst 17.3% Tran’s individuals in this study included COVID-19, HBV, HCV, HIV, TB, and typhoid. Only 0.9% of the sample was HIV positive as compared to a study held in Pune, India where a massive burden of HIV was prevalent with more than 45% of Hijras being positive for the infection owing to unsafe sexual practices and lack of awareness about the sensitive issue.^15^ Fifty six percent of the participants complained of depression, 59% anticipated anxiety and 26% reported suicidal thoughts. In a study conducted in Islamabad and Rawalpindi, Pakistan, the prevalence of suicide ideation among transgender was high (38.6%) majorly due to depression and anxiety in this community however, suicide attempted rate was 18.5%.^16^ Insights arising from this study reported 34% of the respondents feeling isolated from society while a large proportion 89% of our sample felt discriminated by the fellow citizens.76% of the respondents admitted being physically assaulted. Virginia Transgender Health Initiative Survey (THIS) notified a 37% attack rate upon trans men and transwomen since the age of 13.^17^

Amongst the selected sample of this study, 86% of the transgenders were sexually active as compared to a study retrieved from PUNE transgender community where 84% of transgenders were involved in sexual activities.^15^ In this study 77% of the sample was sexually active, only 17% were aware of the STDS while 2.8% complained of genital warts, 2.3% reported discharge, 21% had genital ulcers and 76% of the participants have been screened for HIV. In another study conducted in Pakistan 58% of Hijras had at least one and 38% had multiple STIs.^18^ In this study, 74% of the transgenders received money for their services and 77% mentioned the use of condoms to prevent the risk of acquiring STDs. Gender discrimination and transphobia affect educational and employment opportunities for transgender women in the region leading to the exchange of money or goods for sex.^19^ In another study conducted in Pakistan, few transgenders used condoms. Most (94%) could identify a condom, but 42% reported never needing one.^20^

Eighty five percent of the respondents, in this study visited doctors for consultation, 6.1% visited nurses. Government hospitals were the most frequently selected medical setting by 70% the trans community and only 18% afforded treatment at private hospitals. An investigation spanning across seven cities in India explored the gender transition facilities being offered at private and public hospitals. Findings revealed a near absence of such services at public hospitals while an unexceptionally high treatment cost in private sector remained an important factor in avoidance of seeking professional care. Hence, these trans genders chose unqualified personals for their treatment.^21^ Unfortunately, 11.5% of this sample chose not to visit the hospital settings even when needed. Half of the sample complained of non-acceptance by the health care providers, 29% felt ashamed of their identity, 17% could not afford the treatment and 4% didn’t have a CNIC card. Argentina enjoys a worldwide recognition for promoting transgender healthcare. However, in a study conducted to explore the reasons for avoiding treatment, 40.7% of the sample were embarrassed to reveal their trans identity. Deep rooted mistreatment, discrimination by professionals heightened the associated stigma further.^22^ In this study, only 48% of the respondents had a routine medical checkup in the past two years. Comparatively, in another study conducted in 2013, 364 transgenders of Massachusetts answered a digital web-based survey online. Amongst those participants, 23.6% reported being unable to access health care in the past 12 months.^23^

Eighty one-point eight percent of the sample confessed that, they felt neglected in terms of seeking treatment and 79% were segregated by the health care provider. In addition to this, transgenders repeatedly encounter health care providers who delay the service or bluntly deny to provide them with adequate treatment and care. A study conducted in Iran and Turkey revealed that the transgender population in these countries also faces healthcare stigma, a lack of providers with competence in caring for transgenders, and discrimination related to gender-discordant identity documents.^24^ In this study 84% of the respondents believed that there was lack of trans specific health education and knowledge of health care professionals. Same issue has been highlighted by Somro MH in Pakistan.^25^ Such studies are limited due to lack of access to data and hinderances to reach the marginalized population.

### Limitations of the study:

Major limitation of this study was that data was collected only from Lahore so results cannot be generalized to whole transgender population of Pakistan. Researchers were granted access to transgender community through snow-ball sampling technique with the help of gurus so probability of inclusion of all in the sample was reduced. On the other hand, inclusion of a large sample size is a strength of this study. For better understanding of the mentioned topic, such studies should be replicated in other provinces and cities of Pakistan.

## CONCLUSION

This study gives us an insight of health problems faced by transgenders socially, physically, mentally and the barriers faced by them in accessing healthcare. The main health problems of transgender are diabetes, hypertension, hepatitis C, depression and genital tract ulcers. Majority of the transgenders are sexually active, amongst whom a high proportion had knowledge about sexually transmitted diseases and usage of condoms. Seventy percent of the transgender population went to government hospitals. About 70% trans-genders receive poor quality of health care services. Non acceptance, feeling ashamed, non-availability of CNIC and non-affordability have been reported as major barriers in getting desired health care.
